# Computed Tomography-Guided Percutaneous Radiofrequency Ablation of the Splanchnic Nerves as a Single Treatment for Pain Reduction in Patients with Pancreatic Cancer

**DOI:** 10.3390/diagnostics11020303

**Published:** 2021-02-13

**Authors:** Stavros Grigoriadis, Maria Tsitskari, Maria Ioannidi, Periklis Zavridis, Ioannis Kotsantis, Alexis Kelekis, Dimitrios Filippiadis

**Affiliations:** 1 2nd Department of Radiology, Medical School, National and Kapodistrian University of Athens, University General Hospital “ATTIKON”, 12462 Athens, Greece; ioannidimary@gmail.com (M.I.); akelekis@med.uoa.gr (A.K.); dfilippiadis@yahoo.gr (D.F.); 2 Interventional Radiology Department, George Papanikolaou General Hospital, 57010 Thessaloniki, Greece; mariadote@hotmail.com (M.T.); pzavridis@gmail.com (P.Z.); 3 Section of Medical Oncology, 2nd Department of Internal Medicine, National and Kapodistrian University of Athens, Attikon University Hospital, 12462 Athens, Greece; ikotsantis@gmail.com

**Keywords:** splanchnic nerves, neurolysis, radiofrequency, computed tomography, pain

## Abstract

The aim of this paper is to prospectively evaluate the efficacy and safety of percutaneous computed tomography (CT)-guided radiofrequency (RF) neurolysis of splanchnic nerves as a single treatment for pain reduction in patients with pancreatic cancer. Patients with pancreatic ductal adenocarcinoma suffering from abdominal pain refractory to conservative medication who underwent CT-guided neurolysis of splanchnic nerves by means of continuous radiofrequency were prospectively evaluated for pain and analgesics reduction as well as for survival. In all patients, percutaneous neurolysis was performed with a bilateral retrocrural paravertebral approach at T12 level using a 20 Gauge RF blunt curved cannula with a 1cm active tip electrode. Self-reported pain scores were assessed before and at the last follow-up using a pain inventory with numeric visual scale (NVS) units. The mean patient age was 65.4 ± 10.8 years (male-female: 19-11). The mean pain score prior to RF neurolysis of splanchnic nerves was 9.0 NVS units; this score was reduced to 2.9, 3.1, 3.6, 3.8, and 3.9 NVS units at 1 week, 1, 3, 6, and 12 months respectively (*p* < 0.001). Significantly reduced analgesic usage was reported in 28/30 patients. Two grade I complications were reported according to the Cardiovascular and Interventional Radiological Society of Europe (CIRSE) classification system. According to the results of the present study, solely performed computed tomography-guided radiofrequency neurolysis of splanchnic nerves can be considered a safe and efficacious single-session technique for pain palliation in patients with pancreatic ductal adenocarcinoma suffering from abdominal pain refractory to conservative medication. Although effective in pain reduction the technique seems to have no effect upon survival improvement.

## 1. Introduction

Patients with pancreatic ductal adenocarcinoma will complain of abdominal pain at some stage during the disease’s course; abdominal pain in these patients is multifactorial and its pathophysiology includes either a mixture of neuropathic, visceral, and somatic mechanisms or can be due to side effects of medication and other treatments [1]. This pain is disabling and associated with depression, disturbed sleep, and fatigue, and it can serve as an outcome and survival predictor [1,2,3].

Therapeutic armamentarium for abdominal pain in patients with pancreatic ductal adenocarcinoma includes pancreatic enzyme replacement therapy, chemotherapy, and analgesic therapy, according to the “WHO analgesic ladder” and neurolytic techniques [1,4]. Neurolytic techniques include chemical ones by means of ethanol or phenol injection as well as thermal ones by means of radiofrequency or cryoablation application [5]. Neurolysis targets in patients with pancreatic ductal adenocarcinoma include celiac plexus or splanchnic nerves either solely performed or in single-session combined approaches [5,6,7,8,9,10]. Furthermore, neurolysis of splanchnic nerves can be performed in patients not responsive to celiac plexus neurolysis [11]. Radiofrequency neurolysis has a shorter risk-benefit ratio compared with alcohol neurolysis since it is a more sophisticated and targeted interventional technique; when compared to medical management by opioids percutaneous neurolysis excels in terms of fewer burdensome side effects [7].

The purpose of this study is to evaluate the efficacy and safety of percutaneous computed tomography (CT)-guided neurolysis using continuous radiofrequency as a single-session palliative technique solely performed in patients with pancreatic ductal adenocarcinoma with abdominal pain refractory to standard treatments proposed in the WHO three-step analgesic ladder.

## 2. Materials and Methods

### 2.1. Patient Selection and Evaluation

The present study is a prospective observational study evaluating patients with pancreatic ductal adenocarcinoma suffering from abdominal pain refractory to conservative medication treated by CT-guided neurolysis of splanchnic nerves by means of continuous radiofrequency. The primary objective was pain reduction at the end of the follow-up period. Secondary objectives included reduction of analgesics uptake and overall survival evaluation. Inclusion criteria included patients ≥18 years old with pancreatic cancer, coagulation parameters within normal limits, and a life expectancy of >3 months. Exclusion criteria included non-compliance of patients, uncontrollable international normalized ratio (INR), systematic or local infection, expected survival less than 3 months, an Eastern Cooperative Oncology Group (ECOG) score less than 3, and presence of a medical or psychiatric illness that would preclude informed consent or follow-up. Each patient underwent laboratory coagulation tests at least 24 h prior to the percutaneous neurolysis session. The patients were fully informed about the procedure, the possible complications, and the medical alternatives available; informed written consent for both the technique and the study was obtained in all cases. Patient characteristics, radiofrequency technique, efficacy, and complications were evaluated.

### 2.2. Percutaneous Radiofrequency Neurolysis

**Figure 1:**
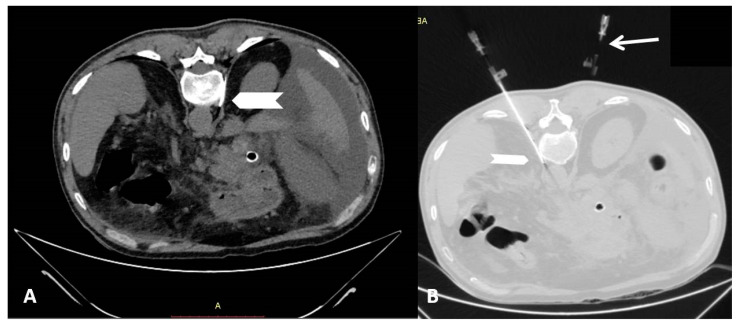
A 60-year-old, male patient with pancreatic ductal adenocarcinoma suffering from abdominal pain refractory to conservative medication was treated by CT-guided neurolysis of splachnic nerves by means of continuous radiofrequency. (**A**) Computed tomography axial scan (soft tissue window) illustrating the curved RF needle/electrode (white chevron) retrocrurally and anterolaterally to the T12 vertebral body on the right side. (**B**) Computed tomography axial scan (lung window) illustrating the curved RF needle/electrode (white chevron) retrocrurally and anterolaterally to the T12 vertebral body on the left side. Additionally, the vein catheters (white arrows) used for skin entrance of the blunt RF needle/electrode are illustrated as well.

Radiofrequency neurolysis of splanchnic nerves was always performed in an inpatient setting. Computed tomography guidance with sequential scanning (120 Kv peak, 240 mAs wavelength, and 0.9 mm slice thickness) was used for planning, targeting, and intra-procedural modification during the session. A combination of local anesthesia with lidocaine hydrochloride 2% and intra-venous analgesia with paracetamol was used to treat intra-procedural pain [12]. The access route is similar to what is performed with other neurolytic techniques such as alcohol injection. Under local sterility, neurolysis was performed with a percutaneous posterior paravertebral approach in all cases with 15 cm/20 G radiofrequency electrodes with curved blunt 1 cm active tips (Equip Medikey BV, Gouda, The Netherlands) being placed bilateral and anterolaterally to the T12 vertebral body. After the initial CT scan, the skin entry point was selected and two vein catheters were inserted. Coaxially the radiofrequency needle/electrode was inserted on each side at the level of interest and its approach was evaluated with sequential CT scans. Injection of 1–3 cc of iodinated contrast medium was used to verify retrocrural and extravascular final placement of the needle electrode (Figure 1). Once in the correct location, a neurolysis session was performed (2 cycles of 85° for 90 s each).

### 2.3. Outcome Measures

Computed tomography assessed potential immediate complications during and at the end of the neurolysis session. The patients remained in the hospital overnight and were then discharged. We evaluated technical success, treatment response (clinical success), and complication rates. Technical success was defined as successful radiofrequency electrode placement at the level of interest on both sides. Clinical success was defined as pain reduction (>4 pain score units) as recorded in the numeric visual scale (NVS) pain scores. The definition of complications was assigned according to the Cardiovascular and Interventional Radiological Society of Europe (CIRSE) classification system [13].

### 2.4. Statistical Analysis

Quantitative variables were expressed as mean values (SD), while qualitative variables were expressed as absolute and relative frequencies. Repeated measurements analysis of variance (ANOVA) was adopted to evaluate the changes observed in pain levels over the follow-up period. Bonferroni correction was used in order to control for type I error. To longitudinally assess changes in pain, mixed linear regression models were fit that account for multiple measurements per individual obtained at different time points. All analyses were conducted using a random coefficient model with the intercept being random and a covariance structure of variance components. Gender and age along with their interaction with time were also tested in the models. All reported p values are two-tailed. Statistical significance was set at *p* < 0.05 and analyses were conducted using SPSS statistical software (IBM Corp. Released 2015. IBM SPSS Statistics for Windows, Version 23.0. Armonk, NY, USA).

## 3. Results

The sample consisted of 30 participants with a mean age of 65.4 years (SD = 10.8 years) and a median age of 67 years (Table 1). Most of the participants were males, with the percentage being 63.3%. Technical success was 100% (i.e., electrode placement at the level of interest on both sides was successful in all patients). There was no need for hydrodissection or any other ancillary methods.

**Figure 2:**
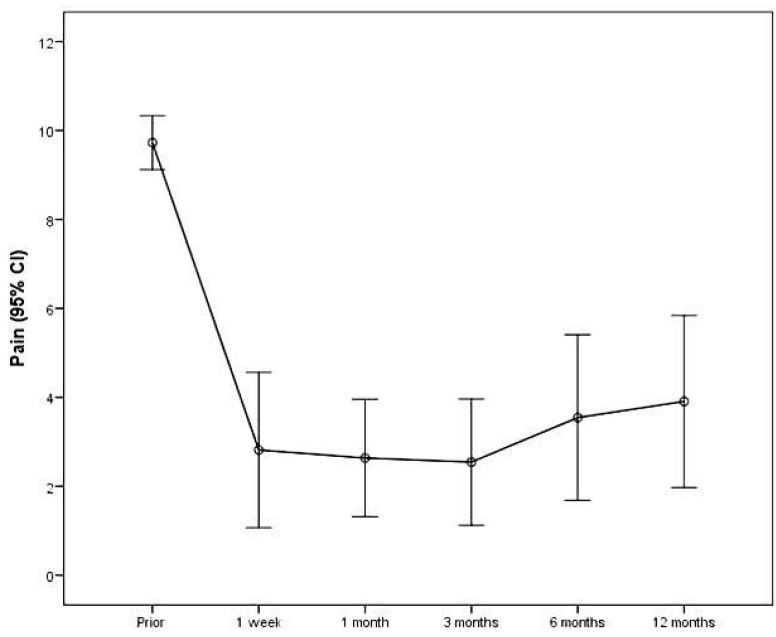
Participants’ pain levels throughout follow-up.

The pain measurements are presented in Table 2. Mean change from prior measurement to 1 week after was significant and equal to −6.0 (SD = 2.7) (*p* < 0.001). The changes between the following consecutive measurements were not significant (*p* > 0.05). Overall mean change from prior measurement to 12 months was significant and equal to −5.8 (SD = 2.8) (*p* < 0.001). Pain was significantly diminished throughout the follow-up period (β = −0.01; SE = 0.003; *p* = 0.043). Furthermore, it was found that pain decreased significantly from prior to the first week measurement (β = −0.85; SE = 0.06; *p* < 0.001) and then it remained at a similar level until 12 months after (β = 0.005; SE = 0.003; *p* = 0.127) (Figure 2).

Participants’ pain measurements descriptive statistics are presented in Table 3 by gender and age. Using mixed models, the interaction of time with gender was not significant (*p* = 0.479), indicating a similar pain decrease in both genders. Similarly, the interaction of time with age was not significant (*p* = 0.098), indicating a similar pain decrease in both age groups. Pain level decreased in all subgroups. Significantly reduced analgesic usage was reported in 28/30 patients.

Two cases of pneumothorax requiring nothing but observation and not prolonging the programmed hospitalization (Grade I) were reported according to the CIRSE classification system.

As far as follow-up is concerned, all patients were evaluated for pain reduction at 3 months post neurolysis while 13 and 19 patients had died due to disease progression at 6 and 12 months follow-up, respectively.

## 4. Discussion

The present study adds to the growing number of case series showing that percutaneous neurolysis of splanchnic nerves is an efficacious and safe technique for pain reduction in symptomatic patients with pancreatic ductal adenocarcinoma who complain of abdominal pain refractory to analgesics and other medications [11,14,15,16]. Comlec et al. evaluated 34 patients with pain due to pancreatic cancer who were non-responders to celiac plexus neurolysis, reporting 71% pain reduction at 3 months follow-up [11]. Shwita et al. compared retrocrural celiac plexus versus splanchnic nerve block reporting clinically comparable efficacy of the two approaches [14]. Amr et al. compared radiofrequency ablation (30 patients) and chemical neurolysis (30 patients) of splanchnic nerves for the management of abdominal cancer pain, concluding that the radiofrequency arm provided faster and longer-lasting pain reduction effect with a better safety profile in more patients than the alcohol group [15]. In the present study, there was a statistically significant decrease of ~6 NVS units from baseline to 3 months follow-up which lasted until 12 months in the surviving patients.

A retrospective propensity score matching analysis by Oh et al. reported that celiac plexus neurolysis, although efficient for pain reduction, did not affect survival for patients with unresectable pancreatic cancer [17]. Similarly in the present study, although survival was not included in the evaluated objectives, it has to be noted that from the 30 patients undergoing splanchnic nerves neurolysis at baseline and evaluated till 3 months follow-up only 17 and 11 were still alive at 6 and 12 months follow-up, respectively. In all the deceased patients, disease progression was the cause of death.

Complications of celiac plexus neurolysis include among others hypotension, diarrhea, and neurological injury [5,18,19]. Neurolysis of splanchnic nerves seems to be governed by a higher safety profile when compared to celiac plexus approaches [14]. In the present study, there were two technically oriented complications (pneumothorax due to inadvertent lung puncture requiring nothing but observation and not prolonging patient hospitalization) while there were no clinically oriented post-neurolytic complications. Alternative approaches for splanchnic nerves neurolysis have been described including trans-discal ones [20]. In the present study, a retrocrural approach with needle placement anterolateral to the T12 vertebral body was technically feasible in all sessions with no need for hydrodissection or any other ancillary technique.

Direct percutaneous ablation of pancreatic carcinoma under imaging guidance has been shown to result apart from local tumor control to pain reduction and quality of life; up until now, there are no reports published concerning the comparison of tumor ablation versus neurolysis aiming for pain palliation [21,22].

Limitations of the present study include the small number of participants; the lack of comparison to a group of patients undergoing alternative (surgical or another type of neurolysis by means of alcohol or cryoablation) approaches for pain reduction.

In conclusion, according to the results of the present study, computed tomography-guided radiofrequency neurolysis of splanchnic nerves solely performed can be considered a safe and efficacious single-session technique for pain palliation in patients with pancreatic ductal adenocarcinoma suffering from abdominal pain refractory to conservative medication. Although effective in pain reduction the technique seems to have no effect upon survival improvement.

## Figures and Tables

**Table 1:** Age and gender of the participants.

	N (%)
Gender	
Females	11 (36.7)
Males	19 (63.3)
Age, mean (SD)	65.4 (10.8)
Age	
<67	15 (50.0)
>67	15 (50.0)

**Table 2:** Participants’ pain levels throughout follow-up.

Pain	N	Mean (SD)	Mean Change from Previous Measurement (SD)	P^+^
Prior	30	9.0 (1.4)	-	
1 week	30	2.9 (2.2)	−6.0 (2.7)	<0.001
1 month	30	3.1 (2.1)	0.2 (2.3)	1.000
3 months	28	3.6 (2.3)	0.4 (1.4)	1.000
6 months	17	3.8 (2.7)	0.6 (1.4)	0.869
12 months	11	3.9 (2.9)	0.4 (0.9)	1.000
Mean change from prior to 12 months (SD)			−5.8 (2.8)	<0.001

**Table 3:** Participants’ pain levels throughout follow-up, by gender and age group.

Pain	Gender		Age	
	Females	Males	<67	>67
	Mean (SD)	Mean (SD)	Mean (SD)	Mean (SD)
Prior	9.2 (1.5)	8.8 (1.5)	8.9 (1.6)	9.0 (1.4)
1 week	2.7 (2.4)	3.1 (2.2)	2.9 (2.2)	3.0 (2.4)
1 month	3.3 (2.5)	3.0 (1.9)	3.5 (2.1)	2.7 (2.0)
3 months	3.6 (2.8)	3.6 (2.0)	4.2 (2.2)	3.2 (2.4)
6 months	4.0 (3.0)	3.6 (2.7)	4.8 (3.2)	2.9 (1.9)
12 months	2.3 (1.5)	4.9 (3.1)	4.9 (3.1)	2.3 (1.5)
Changes:				
From prior to 1 week	−6.5 (3.2)	−5.8 (2.4)	−6.1 (2.7)	−6.0 (2.8)
From 1 week to 1 month	0.5 (1.9)	−0.1 (2.5)	0.7 (2.5)	−0.3 (2.0)
From 1 month to 3 months	0.4 (1.5)	0.5 (1.3)	0.3 (1.5)	0.5 (1.2)
From 3 to 6 months	0.3 (1.8)	0.7 (1.6)	0.9 (1.7)	0.3 (1.0)
From 6 to 12 months	0.0 (0.0)	0.6 (1.1)	0.6 (1.1)	0.0 (0.0)
From prior to 12 months	−7.8 (1.5)	−4.7 (2.98)	−4.7 (2.9)	−7.8 (1.5)

## Data Availability

The data presented in this study are available on request from the corresponding author. The data are not publicly available at the moment since the present study is the basis for the Ph.D. thesis of the corresponding author.
